# Supplementary figures and images for: Induction of a transcriptional adaptation response by RNA destabilization events
Source: EMBO Rep. 2025 Mar 24;26(9):2262–79. doi: 10.1038/s44319-025-00427-3 (PMC12069562; doi:10.1038/s44319-025-00427-3)

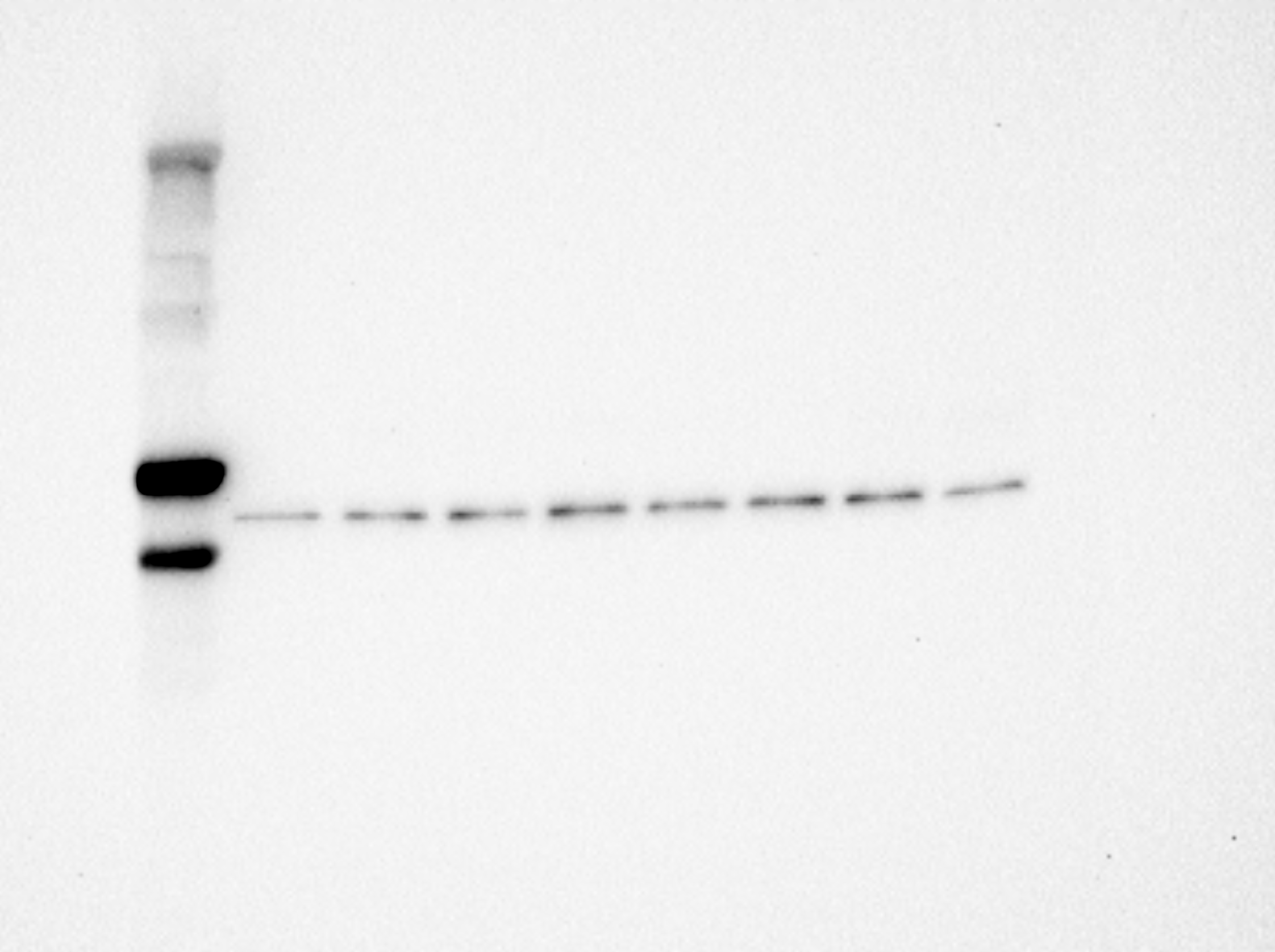

Supplement: Supplementary file 4 — Source data Fig. 2 [file 44319_2025_427_MOESM4_ESM.zip › Figure 2/Figure 2B/Actg1.tif]

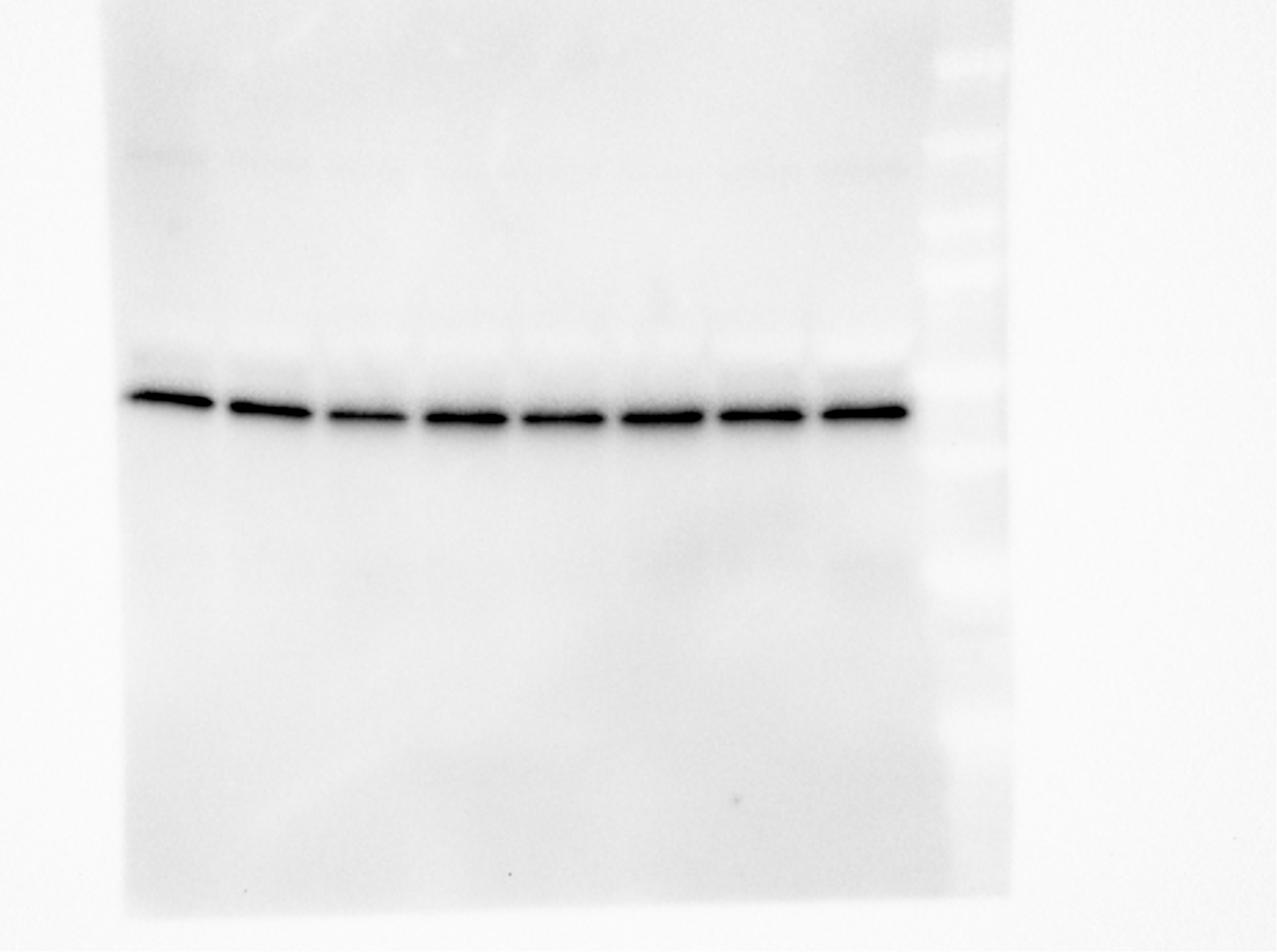

Supplement: Supplementary file 4 — Source data Fig. 2 [file 44319_2025_427_MOESM4_ESM.zip › Figure 2/Figure 2B/gamma_Tublin.tif]
