# Supplementary material for: Induction of a transcriptional adaptation response by RNA destabilization events
Source: EMBO Rep. 2025 Mar 24;26(9):2262–79. doi: 10.1038/s44319-025-00427-3 (PMC12069562; doi:10.1038/s44319-025-00427-3)
Supplement: Supplementary file 6 — Expanded View Figures [file 44319_2025_427_MOESM6_ESM.pdf]

Expanded View Figures

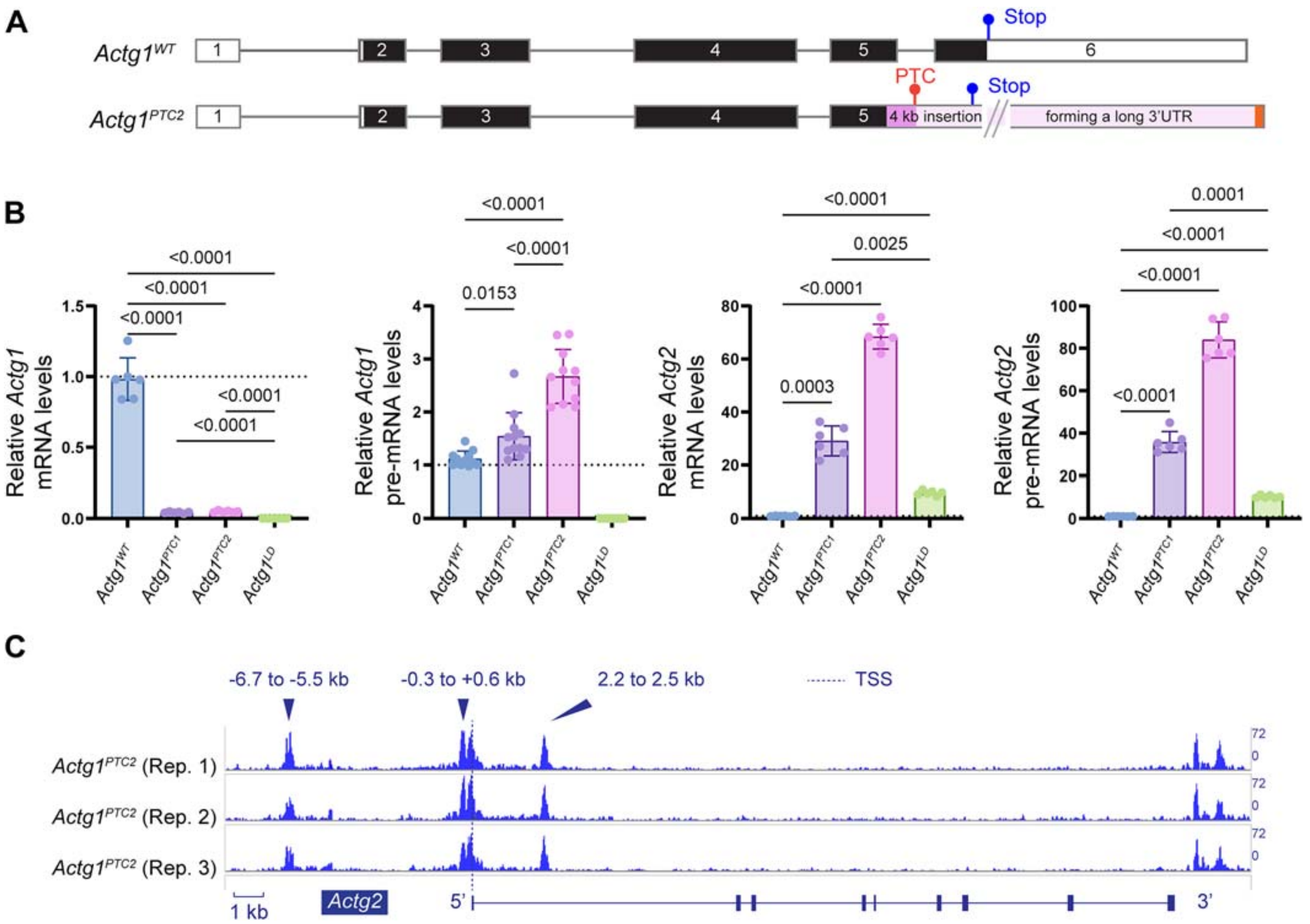

Figure EV1. A second Cas9-induced *Actg1* mutation also leads to mutant mRNA decay and *Actg2* upregulation.

(**A**) Schematic view of $Actg1^{WT}$ and $Actg1^{PTC2}$. Detailed information about the genotype of $Actg1^{PTC2}$ cells can be found in the Materials and Methods section. (**B**) Relative mRNA and pre-mRNA levels of *Actg1* and *Actg2*. $n = 6$–12 biologically independent samples, one-way ANNOVA, pairwise comparison, and exact *p* values are represented in the figure. Data are presented as mean ± standard deviation. (**C**) Chromatin accessibility at the *Actg2* locus. ATAC-seq analysis reveals three open chromatin regions in the $Actg1^{PTC2}$ allele located (1) in the 5′ intergenic region (i.e., −6.7 to −5.5 kb upstream of the transcription start site (TSS)), (2) around the TSS (i.e., −0.3 to +0.6 kb), and (3) in the first intron (i.e., 2.2 to 2.5 kb downstream of the TSS) of *Actg2*; $n = 3$ biologically independent samples.

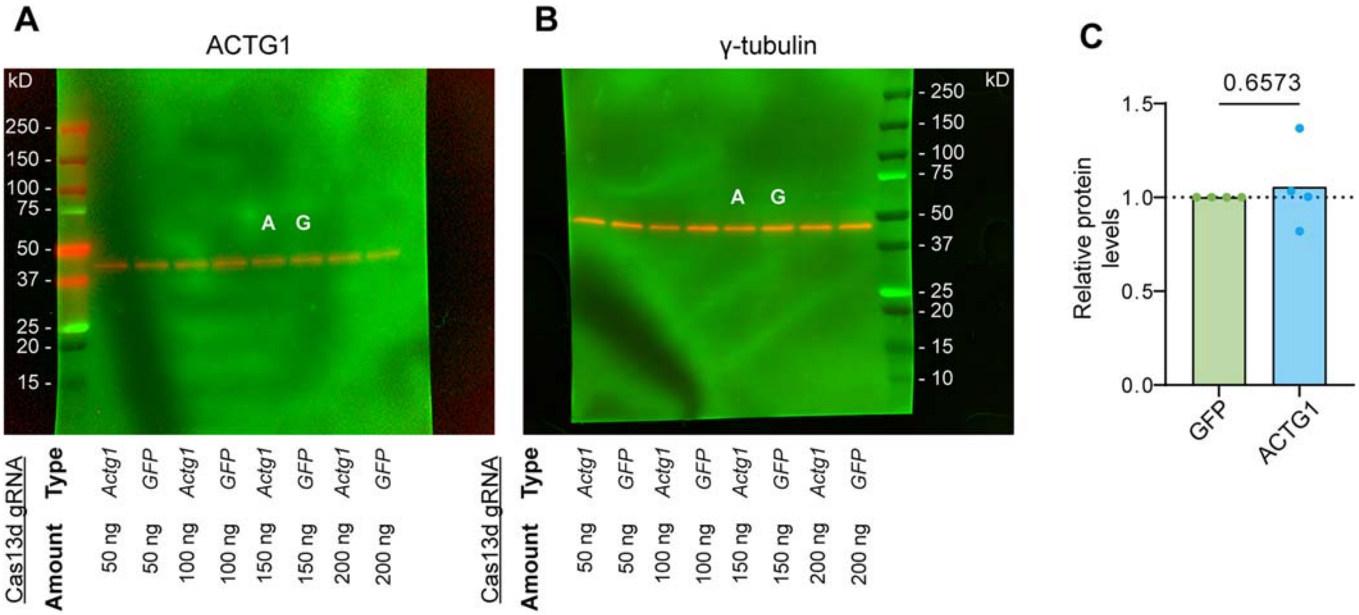

Figure EV2. Cas13d-mediated *Actg1* mRNA cleavage does not lead to a substantial loss of ACTG1 protein.

(**A**, **B**) Western blot analysis for ACTG1 (**A**) and γ-tubulin (**B**) from Cas13d-expressing cells treated with various amounts of *Actg1*- or *GFP*-targeting gRNAs for 14 h; 150 ng of *Actg1* and *GFP* gRNAs were used for the experiments shown in Fig. 2A, B. The molecular weight of ACTG1 is 41,793 Da, and that of γ-tubulin is 51,122 Da. Full uncropped views of blots shown in Fig. 2B; letters A and G on top of the bands in both blots refer to A – *Actg1* and G – *GFP*. (**C**) Quantification of ACTG1 Western blot bands following *Actg1* and *GFP* gRNA experiments; $n = 4$ biologically independent samples, unpaired t-test, and exact p values are represented in the figure.

 © The Author(s)

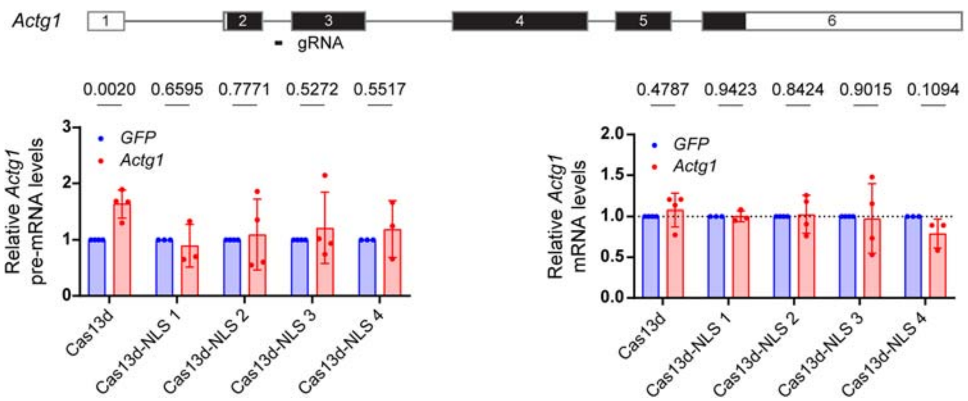

Figure EV3. Targeting *Actg1* pre-mRNA in Cas 13d-NLS cells.

Top: position of Cas13d-NLS gRNA targeting *Actg1* intron 2. Bottom: *Actg1* pre-mRNA targeting does not lead to changes in *Actg1* pre-mRNA (left) or mRNA (right) levels in four independent Cas13d-NLS knock-in clones, compared with cytoplasmic Cas13d-expressing cells transfected with the intron 2 targeting gRNA. $n = 3$–4 biologically independent samples, unpaired t-test, and exact p values are represented in the figure. Data are presented as mean ± standard deviation.

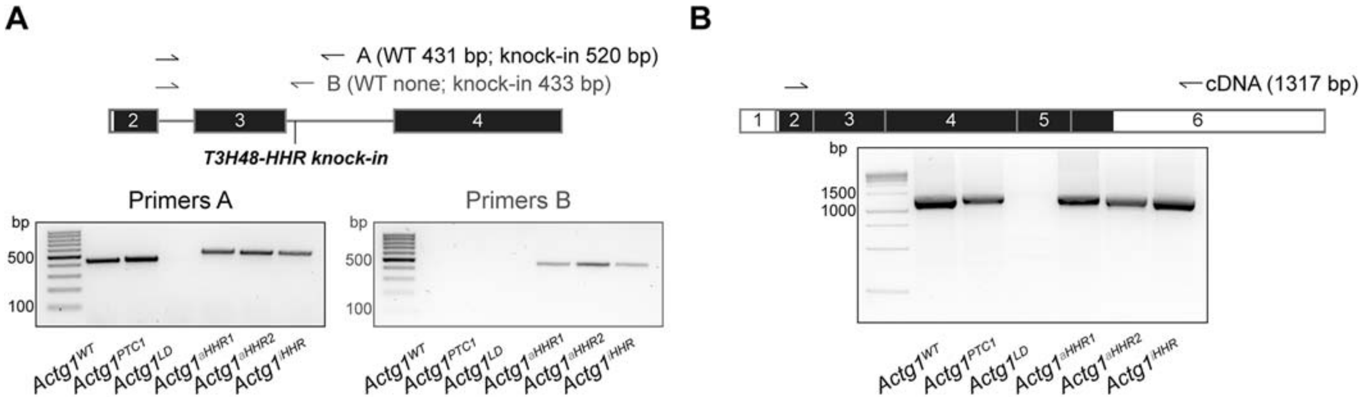

Figure EV4. Validation of the T3H48-HHR knock-in cell lines.

(**A**) Homozygous knock-in of the T3H48 ribozyme in intron 3 of *Actg1* as confirmed by two pairs of genotyping primers in *Actg1*[aHHR] and *Actg1*[iHHR] cells. Two independent clones were generated for *Actg1*[aHHR]. (**B**) *Actg1* cDNA profile in *Actg1*[aHHR] and *Actg1*[iHHR] cells is identical to that in wild-type cells, indicating no alternative splicing caused by the T3H48-HHR knock-in. RT-PCR reactions were run at saturation and therefore, *Actg1* downregulation was not observed in the PTC and T3H48-aHHR alleles in this experiment.

 © The Author(s)
